# Focusing on Resilience and Renewal From Stress: The Role of Emotional and Social Intelligence Competencies

**DOI:** 10.3389/fpsyg.2021.685829

**Published:** 2021-06-24

**Authors:** Han Liu, Richard E. Boyatzis

**Affiliations:** Department of Organizational Behavior, Case Western Reserve University, Cleveland, OH, United States

**Keywords:** emotional and social intelligence competence, resilience development, intentional change theory, resilience, renewal from stress

## Abstract

Individuals are subject to stressful events from daily chronic stress to traumatic life-changing experiences and the resulting impairment. Efforts to reduce stress or stressors are misdirected. Instead, bouncing back or recovering from such experiences, often called resilience is a far more potent way to ameliorate the ravages of chronic stress and move to a state of renewal, thriving and flourishing. Because we infect each other with stress or renewal through emotional contagion, each person's ability to manage their own emotions as well as those of others and their relationships becomes key to health. These capabilities are called emotional and social intelligence. At the trait level, they are personal dispositions but at the behavioral level they are patterns of behavior we call emotional and social intelligence competencies (ESI). This paper is a review addressing the role of emotional and social intelligence competencies in resilience. By focusing on the behavioral level of ESI, designs for more precise research and practical applications as to how to develop ESI and resilience are offered.

## Introduction

Individuals are subject to stressful events from daily chronic stress (Bolger et al., 1989) to traumatic life-changing experiences (Helzer et al., 1987) and the resulting impairment. The ability to bounce back from the ravages of chronic stress and moving to a state of renewal, thriving and flourishing (Boyatzis et al., 2021) has been called resilience. Efforts to reduce stress are misdirected. Instead, we should be focused on increasing resilience and renewal.

Because we infect each other with stress or renewal through emotional contagion, each person's ability to manage their own emotions as well as those of others and their relationships becomes key to health (Daniels and Guppy, 1994; Kay and Merlo, 2020). These capabilities are called emotional intelligence. More specifically, they are called emotional and social intelligence. At the trait level, they are personal dispositions but at the behavioral level they are patterns of behavior we call emotional and social intelligence competencies (ESI) (Boyatzis, 2018).

To facilitate refocusing on resilience and renewal from stress, this paper is a review and conceptualization of the literature on the role of emotional and social intelligence competencies (ESI) in resilience. The contributions will be: (1) synthesizing studies and theories of resilience and ESI to provide a systematic view of the role of ESI in resilience; (2) by focusing on the behavioral level, competencies of ESI, we will be providing practical suggestions as to how to develop ESI and resilience, as well as identifying future research possibilities.

## Stress

There are three major forms of stress: mild or annoying, like your cell phone dropping a call, and the experience of perceived micro aggression, which may be amplified by hypersensitivity and/or events in the larger social environment; acute, like learning a loved one has stage 4 cancer; or traumatic, like being physically in gun violence, riots, and looting. Each form of stress activates the body's Sympathetic Nervous System (i.e., SNS). Traumatic stress events are so profound that they cause repetitive waves of re-experiencing the dramatic activation of the SNS, called post-traumatic distress syndrome. In addition to events occurring to a person, 4 types of internal experience can activate the SNS. They are: (1) when something is important to the person; (2) when outcomes or next events are uncertain; (3) when others are watching or evaluating a person; and uniquely human, (4) when a person anticipates one of the first three (Dickerson and Kemeny, 2004; Segerstrom and Miller, 2004). The accumulation of stress arousals can become “strain” and what physicians call allostatic load.

For most people, at most times in life, it is the cumulative effect of chronic annoying, or mild stress that causes the most damage and impairs cognitive, emotional and perceptual functioning (Sapolsky, 2004; Boyatzis et al., 2021). When the SNS is repeatedly activated, a person's immune system is compromised; neurogenesis is inhibited; blood pressure and pulse rate are increased; breathing gets faster and shallow. On top of all this, a person feels bad. This could result in perceiving events as threatening when, in fact, they are not. Because a person's cognitive and perceptual field is limited during these moments, they are more likely to feel trapped and lose hope for resolution (Bethune and Brownwell, 2010).

Acute stress usually causes a temporary rapid neuroendocrine response (Joëls and Baram, 2009). Cortisol, one of the important markers of stress response, can cause temporary negative impact on memory and attention (Newcomer et al., 1994, 1999). The stress response may be helpful during the event but can also have subsequent negative effects on health (Selye, 1978). A study of 83 undergraduates revealed that people were less persistent with goals when encountered with setbacks after being pre-exposed to acute stress (Bhanji et al., 2016). The study further found out that perception of control can reduce that negative effect.

Acute stress can be caused by short-term episodical stressors with high emotional arousal. Severe single event adversities can be inherently emotional (Kay, 2016). It's essential to find a suitable emotional regulation strategy that can reduce cortisol. Lam et al. (2009) examined 128 participants in a lab research and found that emotional suppression predicted the highest cortisol reactivity during a socially evaluated speech task. Therefore, acute stressors might not directly cause psychological problems unless they are traumatic. However, the wrong response to it and the rumination after it can cause chronic stress. Rumination is often described as a preoccupation or perseveration which is a possible indicator of the person not being able to limit the mental processing of stressful experiences or events (Smith and Alloy, 2009). People with more sophisticated self-regulatory processes can often reframe the experience and let it go.

Traumatic events that are acute life-threatening experiences, such as wars, violent physical assault, natural and man-made disasters (American Psychiatric Association, 2013). Traumatic events can increase risks of multiple psychological problems such as post-traumatic stress disorder (PTSD), acute stress disorder, depression, anxiety, generalized anxiety disorder, and substance abuse (Kessler et al., 1995), and physical problems such as somatic symptoms and physical illnesses (Boscarino, 1996). PTSD in particular is caused by a failure of biological adaptation to the traumatic event in that moment and is less easy to recover from because among other things, a person's cortisol level would be increasing in successive waves of PTSD rather than decreasing and helping the body return to a less aroused state (Yehuda, 2002). Therefore, resilience for traumatic stress should aim at preventing PTSD.

Chronic stress depletes biological, psychological, cognitive and social coping resources for dealing with acute stress and potentially traumatic stress, leading to higher risks of physical and psychological problems (Lepore et al., 1991). Persistent high-level chronic stress can also affect people's response to acute stress, and their subsequent survival and well-being (Gump and Matthews, 1999). Chronic stress in a low social-economic context negatively impacts responses to acute stress (Baum et al., 1999). A study of more than 800 women in Australia found that a higher level of chronic stress predicted a higher level of acute stress, and amplified the negative effects of acute events on depression (Hammen et al., 2009).

## Renewal, Recovery, and Resilience

Given the barrage of stress-inducing events and experiences in life and work, it is fortunate that our human autonomic nervous system has a built-in recovery process, called the Parasympathetic Nervous System (PNS). Activation of the PNS reverses most of the ravages of the chronic SNS activation (Boyatzis et al., 2021). Among the benefits are the increase in functioning of the person's immune system, greater cognitive complexity, creativity and scanning of the environment, neurogenesis, and a sense of well-being (Boyatzis et al., 2021). Although this process has often been called recovery (Sonnentag and Fritz, 2015), it is a process of the body, mind and spirit rebuilding and renewing itself.

The capability of engaging one's own PNS has been called resilience. Resilience is a continuum of adaptation (Hunter and Chandler, 1999; Tusaie and Dyer, 2004). In a broad sense, resilience is needed to rebound from stress, chronic stress and strain (Christensen et al., 2020). Currently, there is an ongoing debate about resilience being a state (i.e., temporary condition occurring whenever the PNS is activated) or an individual disposition (i.e., a personality trait) (Masten and Coatsworth, 1998, Masten and Reed, 2002), or both. The affective mechanism, as important as the other psychological factors in resilience, is much less discussed in resilience literature (Kay and Merlo, 2020). Individual level of resilience, along with cultural and social dynamics, then contributes to group level and collective level of resilience through emotional awareness (Barton and Kahn, 2019) and emotional contagion (Hazy and Boyatzis, 2015).

### Resilience as Trait

Trait resilience, also called ego resilience, is defined as the ability to bounce back from adversities and flexibly adapt to the developmental tasks in the course of life (Block and Block, 1980; Block and Kremen, 1996). Scholars of developmental psychology postulate that resilience is related to the functioning of one's ego. Ego control and ego resilience are the two major functions (Block and Block, 1980; Bowlby, [1969]1997, p. 363) explains that ego resilience is “a person's capacity to modify his level of control according to circumstances.” Ego resilience has both the control element and the flexibility element that is called for by the environment within which one exists.

Ego-oriented psychoanalysis is the basis for viewing resilience as a personal disposition, or trait that describes how individuals gain information from the external environment and respond to it (Lewin, 1935, 1938; Fenichel, 1945). Freud ([1923]2009) once said that the outside world would be a threat to one's ego. But a person's ego develops within an external system (Kegan, 1982). Therefore, ego resilience is both a personality trait that might be transmitted in the womb and possessed at birth, as well as being shaped by one's early experiences (Block and Block, 1980).

Ego resilience is essentially in the process of demonstrating resilient behavior by mediating psychological symptoms, such as anxiety and depression (Philippe et al., 2011). Several personality traits are linked to trait resilience, such as grit (Duckworth et al., 2007), and hardiness (Hodgkinson and Shepherd, 1994; Caza et al., 2020). Ego resilience is also an important predictor of attachment patterns with important caregivers in early life (Bowlby, [1969]1997). A longitudinal study (Arend et al., 1979) showed that higher ego resilience can contribute to a more secure pattern of attachment, i.e., a stable and close relationship with caregivers.

### Resilience as State

Developmental psychologists argue that resilience is a state that is activated by adversity and accomplished by a positive outcome of the process. Luthar et al. (2000) define resilience as a dynamic process that is different from ego resilience. Luthar (2006, p. 742) further points out that resilience is “relatively positive adaptation despite experiences of significant adversity or trauma.” Research on children's development (Garmezy, 1974; Rutter, 1979; Anthony, 1987) focuses on understanding protecting factors and resources that children and adolescents could utilize for their developmental needs. The most common protective factors lie in two categories: self-control and positive relationships (Luthar and Zigler, 1991; Rutter, 1999; Masten, 2001).

Britt et al. (2016) defined *the process* of using resilience as the demonstration of individual resilience capacity. Resilience as a developmental trajectory has two elements: responding to the situation positively and learning from the experience (Caza and Milton, 2011). The developmental perspective suggests that there is a learning orientation in the resilience process (Sutcliffe and Vogus, 2003) that helps individuals integrate learning into their personal development. Caza and Milton (2011) iterates that resilience should encompass the emotional, psychological and behavioral levels. Specifically, the learning of the underlying emotional currents and the subsequent emotional regulation in individuals and groups empower individuals, and thereby their teams and organizations to contain problems with a resilient trajectory (Barton and Kahn, 2019, Kay and Merlo, 2020; Stephens, 2020).

### The Outcome of Resilience

Overall, resilience should result in healthy adaptation at emotional, psychological, cognitive and behavioral levels (Britt et al., 2016). Positive outcome of resilience in children's development refers to behavior that meets the expectation of age and stage appropriate developmental tasks (Masten and Reed, 2002). For adults, there appear to be positive outcomes from resilience at work and in their family relationships (Masten and Reed, 2002).

Post-traumatic growth (Maitlis, 2020) is a form of eudemonic response that might be another possible, positive outcome of resilience. However, posttraumatic growth does not promise a stress-free situation. It might happen while there is still stress (Tedeschi and Calhoun, 1995). Because the growth itself challenges previous beliefs, there will be additional stress from the growth and learning. The need to deal with stress will still be present after the event itself to help with better integration of the learning into one's development of the self.

One example of post-traumatic growth is the possible learning and sense-making in the grief process. Resilience is commonly found in the grief process and can help maintain psychological well-being and help with the later integration of learning through positive transformation of difficult experiences and losses (Bonanno, 2004). Kessler (2019) identifies finding meaning as a sixth stage of the originally five-stage grief process: denial, anger, bargaining, depression, and acceptance (Kübler-Ross and Kessler, 2005). Because of the use of resilience by individuals, many people have successfully transformed their grief into something that is meaningful in their lives (Kessler, 2019).

### Measures of Resilience

Differences in how scholars conceptualize resilience are reflected in the variety of measures used to operationalize resilience in research. A brief review of these measures will illustrate the relative benefits and shortcomings. The common personality trait measures include the ego-resiliency scale (Block and Kremen, 1996) and the Connor Davidson Resilience Scale (Connor and Davidson, 2003). The resilience scale (Wagnild and Young, 1993) and the brief resilience scale (Smith et al., 2008) covers state resilience. There are other personality trait measurements also claim to connect to resilience, such as the hardiness scale (Kobasa, 1979), and the grit scale (Duckworth et al., 2007).

The ego-resiliency scale (Block and Kremen, 1996) measures individuals' consistent capacity to go back to their characteristic level of ego control after adjusting to a situation. The scale measures the dynamic capacity to achieve a system equilibration between the self and the external world through self-control, successful adaptation, and also positive engagement with society (Block and Block, 1980). Because of the dynamic nature of ego-resilience, the scale strives to measure a stable personality trait rather than one time event-specific behavior.

The Connor Davidson Resilience Scale aims to assess resilience in adversities through characteristics such as self-efficacy, sense of humor, patience, optimism, and faith. It also has a focus on solving health-related problems, especially treatment outcomes of anxiety, depression and stress reactions (Connor and Davidson, 2003). Other personality measures such as hardiness scale (Kobasa, 1979) and the grit scale (Duckworth et al., 2007) seem to be measuring aspects of personality that contribute to resilience instead of trait resilience *per se*. It is argued that both the hardiness and the grit scales contribute to physical outcomes of resilience. The hardiness scale predicts resilience more consistently than the grit scale (Caza et al., 2020).

The resilience scale (Wagnild and Young, 1993) measures internal resources that positively contribute to stress resistance and successful adaptation in a difficult situation. It measures state-like characteristics as well as outcomes of resilience rather than a stable trait (Wagnild, 2009; Caza et al., 2020). The scale includes five characteristics: perseverance, equanimity, humor, meaningfulness, and existential aloneness. There are two factors in the scale: acceptance of self and life, and individual competence.

The brief resilience scale measures the state of resilience as the capacity to bounce back (Smith et al., 2008). It is found to be uniquely associated with health, and predicts lower negative health outcomes such as anxiety, depression, negative affect, and physical symptoms (Smith et al., 2008). Smith et al. (2008) also argue that other resilience measures might identify the resources that can facilitate the state of resilience measured in this scale.

### Activities or Experiences of Resilience

There are a wide variety of experiences or activities that have been shown to activate the PNS, and therefore recovery, renewal and resilience (Boyatzis et al., 2021). Whether assuming resilience as a trait or a state, with repeated practice, it can become a habit (Boyatzis et al., 2021). The specific types of activities include (Boyatzis et al., 2021): meditation; yoga; prayer to a loving God; modest exercise; feeling hopeful about the future; being in a loving relationship; helping others less fortunate; having a pet like a dog, cat, monkey or horse; being playful with others and laughing; and a walk in nature. There are other activities and experiences that can elicit or invoke the PNS for individuals, but this brief list has been validated across many populations so are considered generic.

Much debate on resilience in the past has been about the psychological resilience (ego-resilience) and behavior in the process of demonstrating resilience. Recently, scholars have identified resilience at the affective level as a neglected aspect in the process (Kay and Merlo, 2020). Understanding emotions, managing emotions, and one's relationships are critical in the process of fostering resilience (Bion, [1961]2004). Barton and Kahn (2019) argue that understanding the underlying emotions in oneself can lead to more information about interpersonal and group-level dynamics, which gives people more agency, and self efficacy, leading to individual and group level resilience (Kahn, 2001; Barton and Kahn, 2019).

At the heart of each of these experiences is either of two modes: a person managing their own emotional state or building and maintaining better relationships with others (i.e., managing emotions with others). These abilities are called emotional and social intelligence (ESI).

## Emotional and Social Intelligence

Before we explore how emotional and social intelligence (ESI) enables or causes resilient behavior, a brief review of the concept and literature is need. Definitions of ESI is complicated by different measures used in research. ESI is basically a person's ability to manage their own and others' emotions, which includes building and maintaining relationships with others (Boyatzis, 2018). There are four streams of research in ESI (Ashkanasy and Daus, 2005; Boyatzis, 2018). One stream is based on the Mayer, Salovey and Caruso approach to EI as a cognitive ability (Salovey and Mayer, 1990; Mayer and Salovey, 1993; Mayer et al., 1999). They developed a performance ability, the Mayer Salovey Caruso Emotional Intelligence Test (MSCEIT). A second stream are self-assessment measures based on the Mayer-Salovey-Caruso model (Ashkanasy and Daus, 2005). A third stream are a variety of theories of EI. The measures are self-assessment, such as the EQ-i by Bar-On (1997). The fourth stream is based on a variety of theories but assume that ESI are evident in a person's actions and therefore the measures are behavioral assessments, such as the Boyatzis-Goleman Emotional and Social Competency Inventory (ESCI) (Boyatzis, 2018). This measure is an attempt to assess how a person actually handles various emotions. The underlying theoretical models differ in how they were developed. The Mayer et al. model describes emotional intelligence as the ability to discern and regulate emotions (Salovey and Mayer, 1990; Mayer and Salovey, 1993; Mayer et al., 1999). They argue that emotional intelligence consists of four abilities that are moderately interrelated with each other: the ability to perceive, assimilate, understand, and regulate emotions (Mayer et al., 1999). They view EI as closely related to cognitive intelligence which is supported in meta-analyses (O'Boyle et al., 2011). Mayer (2005) proposed a holistic model of personality which included social skills and behavior, but in the most recent publication, Mayer et al. (2016) returned to their earlier description of EI as an ability related to what may be called cognitive intelligence.

The Bar-On Emotional Quotient Inventory (EQ-i) (Bar-On, 1997) started to integrate the emotional and social intelligence in the measurement of EI based on the argument that emotional and social intelligence were closely interrelated personality dispositions and built his theory on the basis of how individuals internally handled their emotions (Bar-On, 2006). The contrast to the MSCEIT (Mayer et al., 2003) and TEIQue (Petrides and Furnham, 2000) was that they intended to assess the ability/trait level aspects of emotional intelligence. As a self-report instrument, the EQ-i is measuring the self-perception and conceptualization of EI and SI. It was not surprising, therefore, when the self-report measures were shown to consistently relate to personality measures in the meta-analyses (Joseph et al., 2015).

A contrasting theory began with the conceptualization of competencies as behavioral patterns with an underlying intent (McClelland, 1973; Boyatzis, 1982; Spencer and Spencer, 1993). Later Goleman (1995, 1998) enhanced the physiological layer to the model, using neuroscience as well as hormonal systems used earlier to explain the physiological foundation for EI and SI. The actual competencies identified emerged from hundreds of inductive studies which began with criterion referencing and selection of the samples on the basis of performance effectiveness in a wide variety of jobs in many countries of the world (Goleman, 1998; Boyatzis and Sala, 2004; Cherniss and Boyatzis, 2013; Boyatzis, 2018) then raised the concept of ESI as competence to study the behavioral level of the construct.

Boyatzis and Sala (2004) contend that ESI should be observable in behavior and relate to real life outcomes (such as job performance). There are two clusters of behavioral ESI: one is the awareness and management of the emotions in oneself to achieve effective outcome; another is the awareness and management of emotions of others to achieve effective or better outcome (Boyatzis, 2018). The most used behavioral EI measure is the Boyatzis-Goleman measure, the Emotional and Social Competency Inventory (ESCI) (Boyatzis, 2018). There are 12 competencies measured, which are grouped in four clusters: self-awareness, self-management, social awareness, and relationship management. The meta-analyses show that these measures have the strongest link to performance among the various types of measures of EI or ESI (O'Boyle et al., 2011; Miao et al., 2016, 2017).

**Figure 1:**
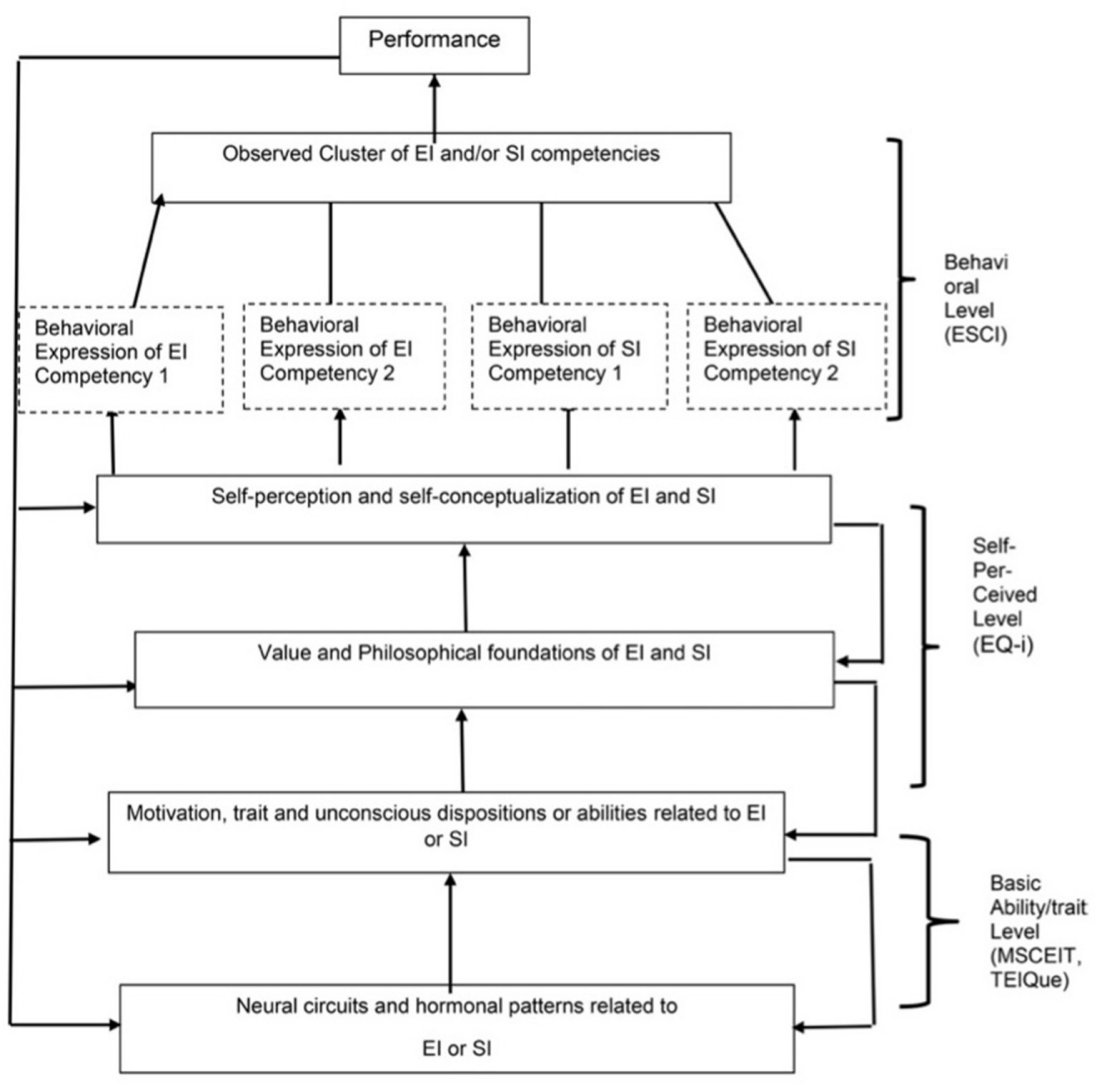
Emotional intelligence (EI) as a multi-level theory [from Cherniss and Boyatzis (2013) which was adapted from Boyatzis (1982, 2009)].

Instead of viewing these different theories and measures as competing directly or pursuing the differential validity of each, it seems more appropriate to claim that they are all defining aspects of EI and ESI. The various measures reflect different levels of the ESI phenomenon operating within the person (Cherniss and Boyatzis, 2013; Boyatzis, 2018). Because of the inductive approach used to identify the behavioral competencies, they may be stronger predictors of many outcomes in both work and non-work settings (Boyatzis, 2018). A summary of the different levels of EI and their measurement is presented in Figure 1.

Because the behavioral level is the most easily changed form of ESI, this paper will use the behavioral level of ESI to illustrate how different levels of the behavioral EI can affect the demonstration of resilience. ESI can both help bring out the behavioral outcomes of resilience and help integrate the reflection from the resilience process to achieve post-event growth.

### ESI Fostering Resilience

As discussed previously, the two major components of resilience and renewal are emotions and the nature of our relationships. Both are at the essence of the use of ESI. Therefore, to understand how ESI may enable and enhance resilience, we need to understand the emotional nature of experiences. Conventionally, resilience is studied in contexts that have negative connotation to one's development, e.g., traumatic experiences, underprivileged family background, hostile environments, and so on. Sudden adversarial events and chronic stressors can both lead to negative emotional experiences. But the nature of renewal and recovery processes require appreciation of positive emotions and how to stimulate them even in the midst of negative events.

As summarized in Table 1, emotional intelligence, at individual level, contributes to state resilience through the use of a variety of ESI competencies and ways to build and maintain quality relationships. For chronic stress, EI enables greater self-awareness while adaptability enables a person to change their approach to a situation. Earlier in this paper, we reviewed a number of activities and experiences that can invoke renewal. Recent research showed that more frequent episodes often of brief duration are needed for renewal from the strain of chronic stress (Boyatzis et al., 2021). The same research showed that a greater variety of activities and experiences also helps improve a person's resilience and renewal.

**Table 1:** Most relevant ESI competencies and activities ameliorating different types of stress.

**Stress type**	**Emotional intelligence**	**Social intelligence**
	Emotional self-awareness and emotional self-management	Managing others and relationship
Chronic	• Using somatic awareness and indicators of one's deep emotional states • Adaptability • Engage in renewal activities multiple times each day preferably in shorter doses to break the cycle of stress hormones • Engage in a variety of renewal activities regularly for distraction	• Caring relationships with others • Helping and coaching with compassion • Shared vision and sense of purpose with others
Acute	• Cognitive reappraisal • Emotional self-control • Active coping strategies for symptom relief • Increase the frequency of renewal activities each day such as deep breathing, meditation, yoga, prayer, etc. to interrupt the cycle of stress hormones • Expand the types or variety of renewal activities	• Caring relationships with others • Helping and coaching with compassion–focus on others • Shared vision and sense of purpose with others
Traumatic	• Cognitive reappraisal • Active coping strategies for symptom relief • Increase the frequency of renewal activities each day such as deep breathing, meditation, yoga, prayer, etc. • Expand the types or variety of renewal activities	• Helping and coaching with compassion–focus on others • Build social support through caring relationships with others

The key to using social intelligence to alleviate chronic stress is for a person to step outside of themselves and focus on others. Research discussed in the next section on using coaching or helping with compassion is focusing on the other person. Using skills like active and deep listening, a person expresses how they care for another or how they share a similar purpose or vision for the desired future (McKee and Massimilian, 2006). Because of emotional contagion, the emotions invoked in these experiences help both the other person and the focal person suffering from chronic stress. Emotional and social contagion travel through the social network: the denser the social network and the, the faster the transmission rate; the higher the emotional intensity of the experience, the faster the transmission rate, like in the social event called Arab spring (Fowler and Christakis, 2008).

For those experiencing acute stress or even traumatic stress episodes, the EI competency of emotional self-control and engaging in active coping for symptom relief enables a person to interrupt the flow of stress hormones and the impairment of cognitive, emotional and perceptual ability that results in resilient state. By increasing the intensity and frequency of renewal activities, a person can combat the higher intensity of the stress reaction due to the acute nature of the stressor (Boyatzis et al., 2021). Cognitive behavioral therapy and affective events theory (Weiss and Cropanzano, 1996) provide further insight into how cognitive reappraisal can help a person perceive a situation differently by reinterpreting it, often as safer or not as hopeless. To engage cognitive reappraisal often requires a degree of emotional self-control to detect the cues emanating during the acute stress episode.

The same uses of social intelligence for chronic stress apply during acute stress as well. Social connections buffer the negative impact of stressful experiences (Cohen and Wills, 1985). Social relationships are important for individuals to cope with chronic stress and acute changes (Kahn, 1998; Dutton and Heaphy, 2003; Wrzesniewski et al., 2003; Yip et al., 2018). Social support, both tangible aid and intangible emotional support, plays a preventative role in traumatic events from PTSD (Bonanno et al., 2007).

By focusing on others and caring for them, a person can step outside of their own intense moment. This provides both momentary relief and distraction, but also invites the other people to show their caring and compassion in return. Whether as part of addressing acute or traumatic stressful events, creating and reminding a person of a social support system, of caring relationships, elicit both hope and compassion, which in turn help to activate the PNS as described earlier in this paper.

Cognitive reappraisal to deal with acute and traumatic stress occurs by changing the perception of the stressful situation, which in turn mitigates the negative impact with less rumination and perceiving the situation as less threatening (Salovey et al., 2002; Gohm et al., 2005).

While all of the above could be applied to invoking state resilience, building trait resilience requires repetitive, behavioral change. Using the same techniques as described for state resilience, but practicing them daily and over time, should help them to become a habit. At the point that using them becomes an unconscious act, the person has begun to improve their trait resilience. Both improving state and trait resilience require sustainable behavioral change.

### Developing ESI and Resilience Through Intentional Change Theory

To develop ESI and resilience, we need to help people change their behaviors, which will lead to trait level change in both ESI and resilience. Intentional change theory (ICT) describes the essential components of desired, sustained change in one's behavior, thoughts, feelings, and perceptions (Boyatzis, 2008). ICT helps people to develop and affirm a personal dream or vision and then to experiment in ways that are internally motivated. The tipping points or triggers to move from one stage of discovery to another is to activate the Positive Emotional Attractor (PEA) more often than the Negative Emotional Attractor (NEA) (Boyatzis, 2008). Stimulating the PEA repeatedly is the approach that can invoke resilience and positive adaptation because it stimulates the neural networks and hormonal systems that allow a person to be open to new ideas and other people (Jack et al., 2013; Boyatzis et al., 2015; Passarelli, 2015).

Change is not a smooth or continuous process. ICT assumes the discontinuous and non-linear nature of change. ICT claims that change is sustained through five important discoveries (Boyatzis, 2008). The five discoveries are: (1) the ideal self and personal vision (find out who I want to be), (2) the real self and the gap between the real self and the ideal self, (3) a learning agenda (identify the gap between the real self and the ideal self), (4) new behavior, thoughts and feelings through experimentation, (5) resonant relationships that help, support and encourage the progress and development in the process.

ICT generates renewal experiences through activation of PNS. With the focus on the ideal self and future vision, people will experience PEA, as opposed to NEA (Jack et al., 2013; Howard, 2015). The work on the real self will then help expand one's self-image to further consolidate the change effort (Taylor, 2006). PEA and NEA are self-regulating states that derive from affect, nervous system arousal and neurological network activation (Boyatzis, 2008). In the PEA sate, people will experience positive emotions that activate PNS (Boyatzis and Cavanagh, 2018). When driven by the ideal self, people will experience hope, efficacy and optimism, which generate positive emotions and activate PNS (Boyatzis and Cavanagh, 2018). Research found that personal vision provides female engineers with more resilient measures to overcome difficulties in technical professions (Buse and Bilimoria, 2014).

ICT sustains change effort by generating self-determined change. Self-determination theory (SDT) postulates that change can be a positive and vital process in which individuals deploy inner and external resources for personality development and behavioral change (Deci and Ryan, 1985; Ryan et al., 1997). To call upon one's inner resources for personality change, SDT highlights the importance of three psychological needs in motivation: competence, relatedness and autonomy (Ryan and Deci, 2000). Feeling of competence, or sense of efficacy, enhances intrinsic motivation when it is combined with a sense of autonomy and social support from connectedness (Deci and Ryan, 1985). According to SDT, individuals are also extrinsically motivated when they identify with certain values through the internalization and integration processes. This way, individuals are motivated to carry out behavior that match personal values. Taylor et al. (2019) theorize that ICT contributes to the sense of competence, relatedness and autonomy through the five discoveries. Subsequently, the sense of competence, relatedness and autonomy will create both intrinsic and extrinsic motivation in individuals. The change effort is then sustained with the intrinsic and extrinsic motivations. Individuals fulfilling the three internal needs through the process enjoy higher level of daily well-being (Sheldon et al., 1996; Reis et al., 2000).

Since 1990, The Weatherhead School of Management at Case Western Reserve University has introduced a course named Leading Change: The Self. It is an MBA course designed to help MBA students to enhance self-knowledge, identify personal vision, and create a developmental plan for achieving that vision. Individuals choose developmental activities according to their own individual situation. They will also receive feedback for them to create a developmental plan. All students are required to finish a personal vision statement and a coaching session about the vision to make sense of it prior to any other activity. They are also required to do a 360-degree feedback assessment and a subsequent coaching session to develop a personal developmental plan. The course has 3 enabling objectives: (a) to systematically identify students' current and desired capability (i.e., knowledge, abilities, values, and interests); (b) to develop an individualized learning agenda and plan for the next 3–5 years; and (c) to explore techniques to assist others in doing the same. This course is based on ICT to develop emotional and social competencies. Boyatzis and Cavanagh (2018) reviewed empirical evidence from 39 longitudinal studies on the course and found that MBA students graduated with dramatic improvement in their ESI competencies both in terms of their self-assessment and their behavior as seen by others. They also found improvement in the two cognitive competencies of system thinking and pattern recognition.

ICT describes not only sustained, and desired individual level of change, but also the group level and organizational level of change. Weick and Roberts (1993) propose that individual actions have impact on the whole system, and that the mindfulness of each other in the system is extremely critical for the whole system to be adaptive to ongoing events and changes. Barton and Kahn (2019) then also build on the idea to explain that individuals' knowledge and awareness of the emotional life in groups create resilient groups that absorb strain brought about by anxiety. This can in turn create a holding environment for groups and organizations to care for their members (Kahn, 1998, 2001).

Emotional contagion exists in groups and organizations (Hatfield et al., 1993, 2018). Emotional contagion may influence people in the same community to act as one (Hazy and Boyatzis, 2015). It happens with positive emotions and negative emotions alike (Barsade, 2002). Coaching individuals with compassion, which is coaching to the PEA, builds the emotional and social intelligence competencies and relationships that can further tip the organization into the PEA state.

## Conclusion

In this paper, we first summarized three different types of stress, which can require both state resilience and trait resilience to bounce back and achieve positive results. We conclude that engaging with positive emotions through activating PEA facilitates state resilience and that dealing with chronic stress can improve state resilience in acute and traumatic stress. Trait resilience can be fostered through repetitive behaviors that constitute habits. Several resilience measures were reviewed. We suggest that a focus on the activation of positive emotion is needed in resilience scales.

Emotional intelligence facilitates state resilience in different types of stress. In order to foster trait resilience, we suggest using Intentional Change Theory to enhance emotional and social intelligence, which engages people with more positive experiences and subsequently being open to cultivating habits through new experiences. We can then facilitate the development of resilience through emotional intelligence using ICT. The ICT-guided design can tip individuals back into positive emotions by focusing on a vision of the ideal self and personal strengths. Sustained changes are found in the results of the courses both at the end of the course and time after the course. More empirical research need to be done outside of the academic setting to verify the positive effect of ESI on resilience using the ICT-guided design. Amidst being besieged by stress, the hope is that we can develop emotional intelligence and resilience. Of course, ESI is not the sole solution to enhancing resilience and renewal, especially when in extreme traumatic events, such as wars, natural disaster, and terrorist attacks. The intensity and frequency of large cultural and social dynamics can also influence resilience at the individual level. For example, the American Psychological Association (2017) reports that 63% of Americans are stressed about mass shootings and future of the country. And for the first time, neither work nor money was the top stressor. Building ESI can amplify the positive effects of a resilient process, especially when ESI is developed through ICT. Therefore, in traumatic situations, ESI can be seen as a protective factor which fosters trait resilience and prevents PTSD. It is more helpful to conduct training for personnel going into work field that might encounter traumatic events in advance.

## Author Contributions

HL and RB contributed to conception and design of the manuscript. HL wrote the first draft of the manuscript. RB revised it critically for important intellectual content. Both authors contributed to manuscript revision, read, and approved the submitted version.

## Conflict of Interest

The authors declare that the research was conducted in the absence of any commercial or financial relationships that could be construed as a potential conflict of interest.
